# Complexity Reduction of Polymorphic Sequences (CRoPS™): A Novel Approach for Large-Scale Polymorphism Discovery in Complex Genomes

**DOI:** 10.1371/journal.pone.0001172

**Published:** 2007-11-14

**Authors:** Nathalie J. van Orsouw, René C. J. Hogers, Antoine Janssen, Feyruz Yalcin, Sandor Snoeijers, Esther Verstege, Harrie Schneiders, Hein van der Poel, Jan van Oeveren, Harold Verstegen, Michiel J. T. van Eijk

**Affiliations:** Keygene NV, Wageningen, The Netherlands; Purdue University, United States of America

## Abstract

Application of single nucleotide polymorphisms (SNPs) is revolutionizing human bio-medical research. However, discovery of polymorphisms in low polymorphic species is still a challenging and costly endeavor, despite widespread availability of Sanger sequencing technology. We present CRoPS™ as a novel approach for polymorphism discovery by combining the power of reproducible genome complexity reduction of AFLP® with Genome Sequencer (GS) 20/GS FLX next-generation sequencing technology. With CRoPS, hundreds-of-thousands of sequence reads derived from complexity-reduced genome sequences of two or more samples are processed and mined for SNPs using a fully-automated bioinformatics pipeline. We show that over 75% of putative maize SNPs discovered using CRoPS are successfully converted to SNPWave® assays, confirming them to be true SNPs derived from unique (single-copy) genome sequences. By using CRoPS, polymorphism discovery will become affordable in organisms with high levels of repetitive DNA in the genome and/or low levels of polymorphism in the (breeding) germplasm without the need for prior sequence information.

## Introduction

SNP discovery is an important area of molecular genetics research aimed at collecting sufficient exploitable sequence polymorphisms to enable high-resolution, high-throughput genotyping at lower costs in the future. However, for many crop species the efficiency of the SNP discovery process is often hampered by the fact that limited amounts of genome sequences are available compared to e.g. *Arabidopsis* and rice, for which draft genome sequences have been completed [1], [2]. Furthermore, the occurrence of (highly) duplicated genome sequences in crops such as maize [3], wheat [4], soybean [5] and pepper [6] impedes conversion of identified polymorphisms into genotyping assays for application in breeding. As a result, available high-throughput SNP genotyping technologies [7]–[10] can not be fully exploited in plant breeding at present due to lack of suitable “content”. This is unlike the situation in humans where several millions of SNPs are known and being utilized in population genetic analysis [11] and medical diagnostics [12]. Hence, there is a need for efficient polymorphism discovery technologies which target unique genome regions in organisms lacking extensive genome sequence information.

The maize (*Zea mays*) genome comprises 2300 to 2700 Mb [13]. Approximately 80% of the total nuclear genome of maize consists of highly repetitive sequences interspersed with single-copy, gene-rich regions. The majority of the repeats are classified as long terminal repeat (LTR)-retrotransposon families that vary in copy number [14]. As a consequence of these genome characteristics, SNP discovery in maize is not straightforward since it is not always obvious how to distinguish a true SNP from sequence differences between duplicated sequences occurring within the genome. Various techniques have been employed to enrich for single-copy sequences in maize, such as High C_o_ *t* selection [15], methylation filtering [16] and hypomethylated partial restriction (HMPR) [17]. HMPR utilizes methylation-sensitive restriction enzymes, thereby relying on the observation that in maize genes often remain unmethylated, whereas most LTR retrotransposons are methylated [18], [19]. Especially HMPR has been shown to be exceptional in depleting retrotransposons to less than 5% [17] of the original content. However, despite the fact that these methods enrich for low-copy sequences, for economical reasons further genome complexity reduction is required to engage in comparative sequencing.

The AFLP® technology [20]–[22] is a powerful DNA fingerprinting technology which has found widespread application in many organisms of diverse origin, including plants, animals, micro-organisms and human. AFLP is based on the selective PCR amplification of restriction fragments from a digest of whole genomic DNA. Its main features are that no prior sequence information is needed and multiplexing levels can be controlled by the choice (and number) of restriction endonucleases and by varying the number of selective bases of the primers used in the amplification process. Besides its many applications as genetic marker technology [22], AFLP is therefore also a robust and scalable method for genome complexity reduction. This feature of the AFLP technology can be exploited to expedite polymorphism discovery by generating in parallel highly similar genome representations of multiple accessions of crop species for high-throughput sequencing.

Here we describe the CRoPS™ technology (acronym for Complexity Reduction of Polymorphic Sequences) and its application in maize. With CRoPS, tagged complexity-reduced libraries of two or more genetically diverse samples are prepared by AFLP, preferably using a methylation-sensitive restriction enzyme. Next, AFLP fragment libraries are sequenced at 5 to10-fold average redundancy in microfabricated, high-density picoliter reactions using the GS system [23]. Resulting sequences are clustered and aligned, and the alignments are mined for SNPs using custom-developed bio-informatics tools. Rigorous quality measures are applied to separate PCR amplification and/or sequence errors from true polymorphisms. The fact that CRoPS is AFLP-based enables its application in many organisms, irrespective of genome complexity and size. The use of homozygous lines in the CRoPS process enables selection of SNPs which are located in low- or single copy genome sequences and therefore have a high conversion rate to genotyping assays for medium to large-scale genotyping.

The CRoPS technology has been applied for polymorphism discovery between the maize lines B73 and Mo17, using AFLP enzyme combination *Hpa*II/*Mse*I. Using a fully automated bioinformatics pipeline we mined more than 1200 high quality putative SNPs and show that 23 out of 30 SNPs were successfully converted into SNPWave assays [24]. We propose CRoPS as a generic approach to significantly enhance polymorphism discovery in vegetable and field crops.

## Results

### GS 20 sequence analysis

After completion of one single GS 20 run, a first bioinformatics analysis was performed using the GS 20 software (i.e. “on-rig” software). A total of 754,199 reads (“totalRawWells”) were obtained. The number of reads after the first filtering for Key sequences (“totalKeyPass”) was 739,042. Of these, 399,252 GS 20 raw sequencing reads remained after the final filtering by the GS 20 software. This number of sequence reads is higher than the specifications of the GS 20 but in line with other runs we performed earlier (data not shown) as well as results reported by others [25]. Their average read length was 103 nt (Table 1).

**Table 1:** Overview of results of one GS 20 CRoPS run in maize

Parameters	Enzyme combination	*Hpa*II & *Mse*I
	Selective bases AFLP primers	A & CT
	Average obtained read length (before trimming)	103 nt
Trimming	Total # of reads after filtering (“GS 20 raw sequencing reads)	399,252
	Reads with sample identification tag assigned	383,566 (96%)
	Faulty reads (no sample identification tag assigned)	15,686 (4%)
	# reads with sample identification tag for sample 1 (B73)	149,226 (39%)
	# reads with sample identification tag for sample 2 (Mo17)	234,340 (61%)
Clustering	Multiple sequence alignments	18,989
	Reads in multiple sequence alignments	211,100
	Average # reads per alignment	11.11
	Singletons	29,141
	# reads in large clusters not contained in MSAs	143,325
Polymorphisms	# putative SNPs	1,225
	# putative indels	37

**Figure 1:**
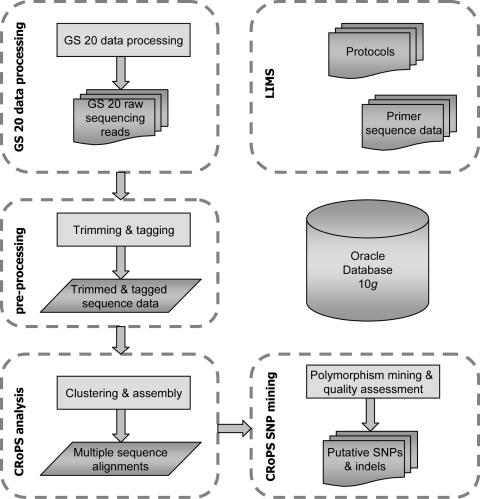
Bioinformatics pipeline for high-throughput analysis of CRoPS sequence runs.

Further bioinformatics analysis took place “off-rig” (i.e. on a separate server) using the CRoPS pipeline (Fig. 1). The GS 20 raw sequencing reads were trimmed (adapter removal) and 383,566 (96%) sequences remained (i.e. sequences for which a significant match with a tagged AFLP primer was found). The reasons for rejection of the remaining 15,686 (4%) reads (classified as faulty reads) were three-fold: 1) AFLP adapter not found, 2) conflict in adapter position (concatamers), and 3) sample identification tag conflict, i.e. a sequence with sample identification tag of one sample at one end and with sample identification tag of the second sample at the other end of the sequence read (so called “mixed fragments”, see further below).

Using the TIGR Gene Indices clustering tool (TGICL) [26], the remaining 383,566 sequences were clustered and assembled. Among these were two very large clusters (119,717 and 23,608 reads respectively) containing heavily repeated sequences. Homology searches using the Basic Local Alignment Search Tool (BLAST) revealed that the sequences within these two clusters were in fact chloroplast sequences. These two clusters were excluded from further processing. Subsequently, sequences within the remaining clusters were assembled into multiple sequence alignments (MSAs) (Table 1). In addition to the 18,989 MSAs containing 211,100 sequence reads, 29,141 (7.6%) singletons were found, i.e. sequences that were not assembled into an MSA.

**Figure 2:**
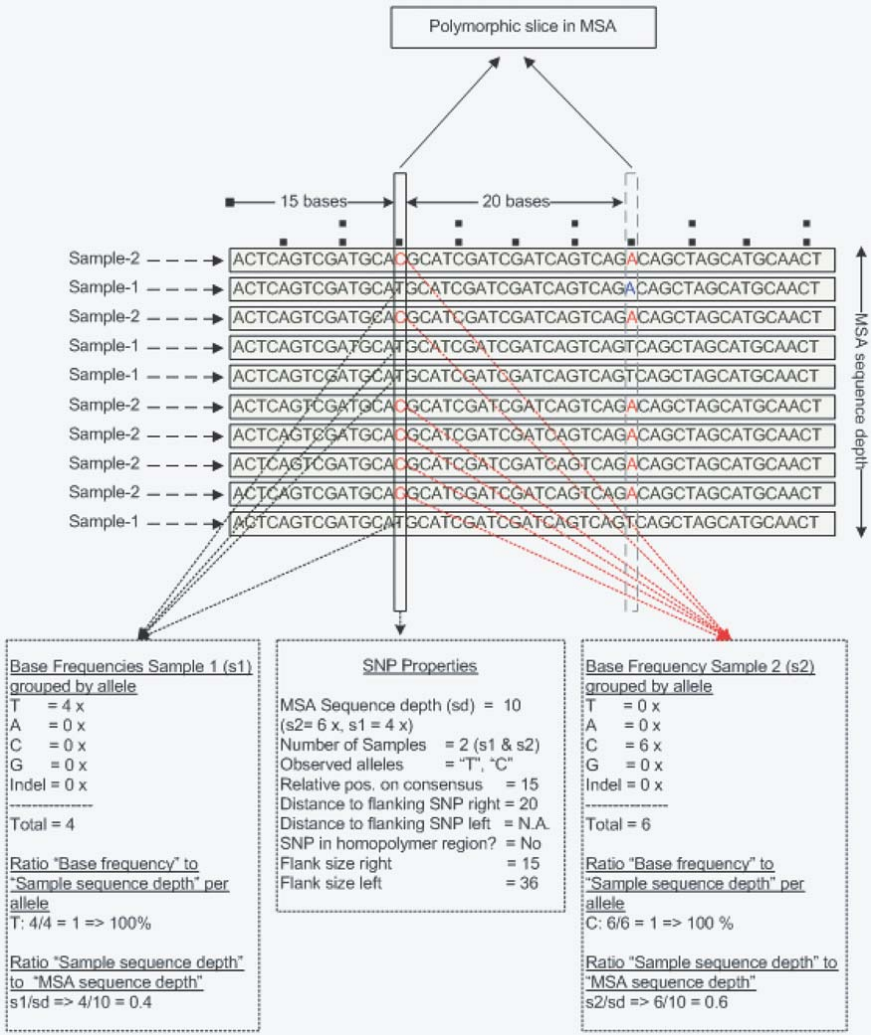
Example of a multiple sequence alignment (MSA) with SNP and sample related properties. SNP properties include sequence depth (sd), the count on the number of reads at the polymorphic position, the relative position of the SNP on the consensus sequence, the distance to the neighboring SNP, flanking sequence size and homopolymeric region information. Sample related properties were derived from the Oracle database. The ratio sample sequence depth to MSA sequence depth is calculated.

Finally, SNPs were mined between the reads contained in an MSA. Parameters for SNP mining were set to include only SNPs for which both alleles were observed at least twice and SNPs not being part of homopolymers larger than 3 bases. The threshold for minimal distance to a neighboring SNP was initially set at one base, i.e. all SNPs were selected irrespective of their distance to (a) neighboring SNP(s). In addition, and importantly, SNPs were mined according to sample origin, i.e. only SNPs “segregating homozygously” between the two maize lines were included. By doing so, a strong filter was created to select against “false” SNPs resulting from alignment of highly homologous duplicated sequences as opposed to genuine SNPs derived from single-copy sequences in the sequenced genome fraction (Fig. 2). As a result, 1262 putative SNPs, including 37 putative indels were mined (Table 1).

### Effect of search parameters on SNP mining

**Figure 3:**
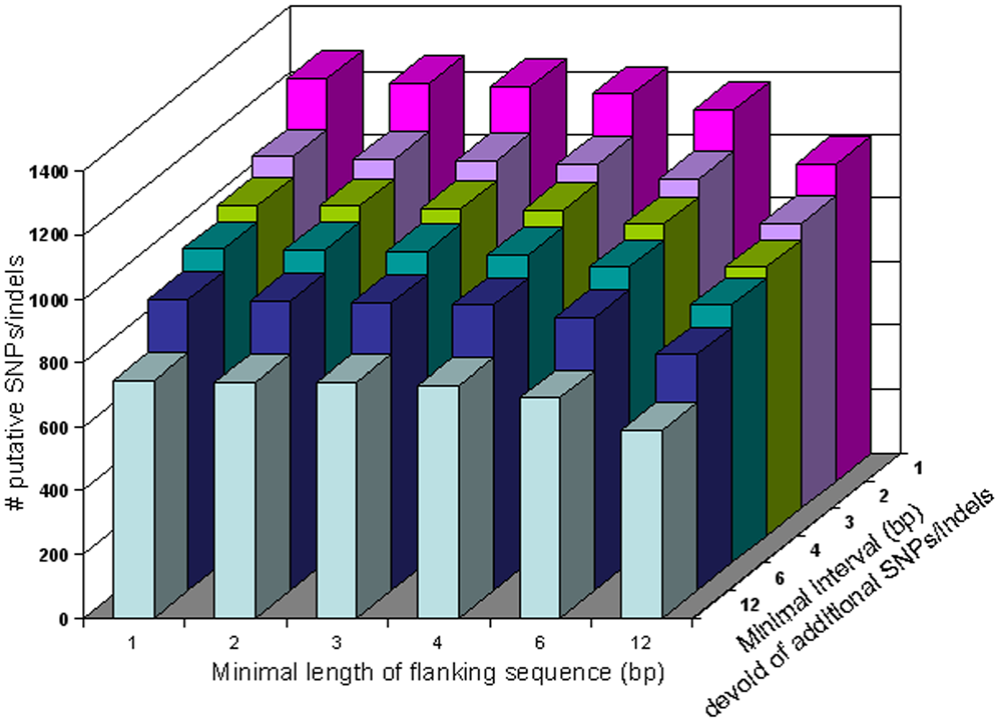
Number of putative SNPs and indels as a function of the minimal length of flanking sequences surrounding the SNP and the minimal interval devoid of additional SNPs/indels.

To investigate the relationship between the number of putative SNPs and SNP mining parameters, SNPs were mined under different parameter settings regarding the minimal available sequence information flanking the target SNP (1, 2, 3, 4, 6 or 12 bases), and the minimal interval of flanking sequence that must be devoid of additional SNPs (1, 2, 3, 4, 6 or 12 bases). SNP mining was performed by varying these two parameters in all 36 (6 times 6) possible combinations, while keeping the other SNP mining parameters, including minimal representation of both alleles at least twice, homopolymer settings and segregation according to sample origin constant. As expected, the number of SNPs mined according to these more stringent criteria decreased to less than 50% (from 1262 to 591; Fig. 3). This selection of 591 SNPs was available for subsequent assay design.

### Validation of putative CRoPS SNPs

**Figure 4:**
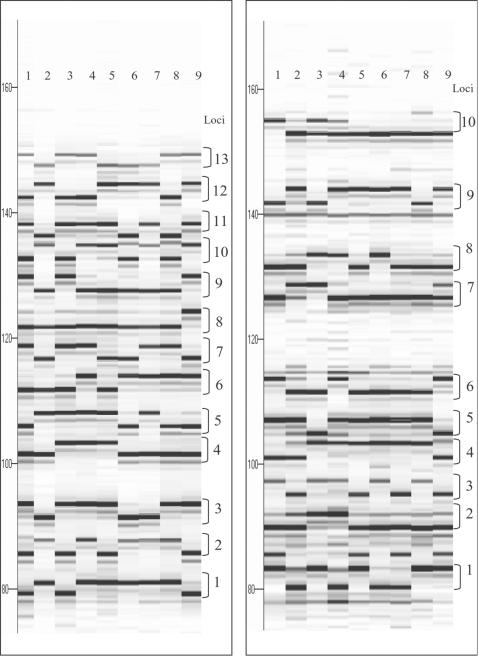
Pseudo-gel image visualizations of two SNPWave assays in maize detected by capillary electrophoresis. Left panel: 13-plex SNPWave assay; right panel: 10-plex SNPWave assay. Number 1-9 represent different recombinant inbred line offspring of B73 and Mo17.

Small-scale validation of putative SNPs was carried out using the SNPWave® technology [24]. From the selection of 591 putative SNPs mined according to the stringent criteria mentioned above (including a minimal of 12 bases flanking sequence surrounding the target SNP and minimal interval of 12 bases devoid of additional SNPs), 30 SNPs were randomly selected. Two 15-plex SNPWave assays were designed and tested using two parental lines and 94 recombinant inbred lines (RIL) offspring of the ISU (B73×Mo17) maize mapping population. For 23 out of 30 tested loci (77%) clear SNPWave reactions products were observed for both alleles, while for the remaining 7 loci one or both alleles were not observed (conversion failure). For all 23 SNP loci functioning properly in the SNPWave assay, the parental lines B73 and Mo17 were polymorphic and segregation was observed among RIL lines (Fig. 4), indicative of a high proportion of mined SNPs being derived from single-copy regions in the genome.

## Discussion

We have applied CRoPS technology for polymorphism discovery in maize and have mined more than 1200 high quality putative SNPs from a single GS 20 sequencing run. We speculate that the stringent but user-definable parameter settings of the bioinformatics pipeline as well as the use of *Hpa*II as one of the restriction enzymes for AFLP template preparation effectively enrich for SNPs located in low-copy or unique genome sequences which have a high success rate of conversion. Since SNPWave is a ligation-based multiplexed SNP genotyping technology [24], we expect conversion rates to be similar when SNPs mined using CRoPS are converted using other ligation-based SNP genotyping technologies [7], [8].

**Figure 5:**
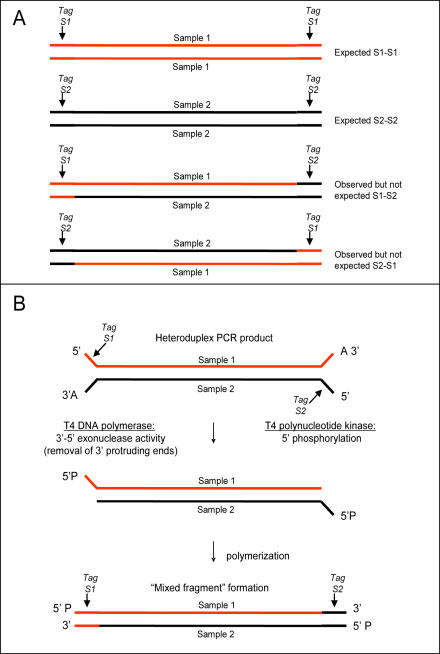
Composition and hypothesized cause of “mixed fragments”. “Mixed fragments” are characterized by the occurrence of the sample identification tag of sample 1 on one side and the sample identification tag of sample 2 on the other side. (A) Schematic representation of observed homoduplex and heteroduplex fragment types containing expected tags and “mixed fragments”. (B) “Mixed fragments” are formed when (1) a heteroduplex is formed between complementary strands of samples 1 and 2, (2) 3′-5′ exonuclease activity of T4 DNA polymerase removes the sequence tags at the 3′ ends, (3) polymerase activity of T4 DNA polymerase extends the 3′ ends using the opposite strand as template, resulting in incorporation of the “wrong” sequence tag, i.e. the observation of “mixed fragments”.

**Figure 6:**
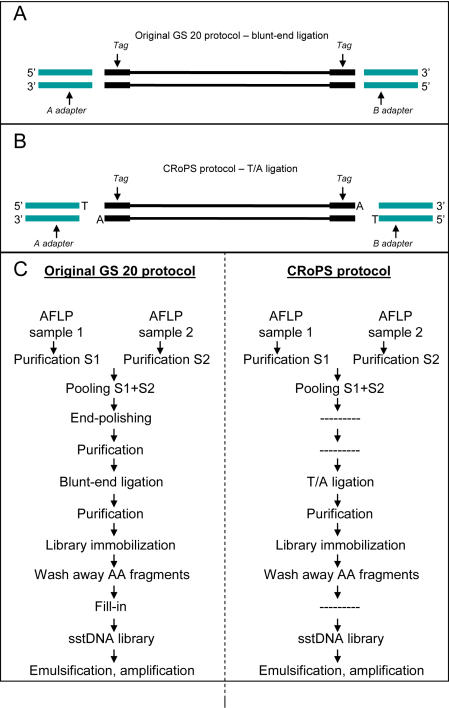
Protocol modification to avoid “mixed fragments”. (A) Blunt-end adapter ligation as per the original GS 20 library preparation protocol. (B) T/A ligation as applied in the CRoPS protocol. Amplification using a polymerase lacking 3′-5′ exonuclease (proofreading) activity is performed resulting in A-addition to the AFLP fragments, after which the T-adapters can be ligated. (C) Flowcharts of the original GS 20 library preparation protocol and the CRoPS library preparation protocol.

During the development of CRoPS, which led to the current sample preparation protocol, we have made several modifications (see Methods) to the original protocol for GS 20 sequencing [23] which was conceived for library preparation of a single sample. These modifications were introduced after the observation of so called “mixed-fragments” in earlier CRoPS runs (results not shown). “Mixed fragments” are sequence reads containing a sample identification tag of one sample at one end and the sample identification tag of another sample at the other end (Fig. 5). In earlier experiments we observed these “mixed fragments” at frequencies between 0.1 and 16% of all obtained reads per run with higher frequencies when more than two samples were involved (data not shown). We suspected that “mixed-fragments” arose from the combination of the enzymatic (3′-5′ exonuclease) mediated recession of free 3′ termini of sample DNA and concomitant fill-in using *Bst* polymerase to create blunt ends for GS 20 adapter ligation as per the original protocol. When this procedure is applied to a mixture of short PCR products containing single-stranded fragments (such as in case of CRoPS), heteroduplex fragments are formed upon mixing the two (or more) samples at this step. Since the different samples contain different four base sample identification tags at their 5′ ends, we suspected that the 3′ ends (which do not match the four base sample identification tags at the 5′ ends of the opposite strand of the heteroduplexes) are removed and filled-in with the opposite strand as template for polymerization. The net result of such an event is a sample identification tag switch (Fig. 5). Therefore, we omitted the end-polishing step and modified the GS 20 adapters A and B by adding a 5′ T nucleotide to allow T/A ligation as commonly performed in PCR product cloning (Fig. 6). This modification was also expected to prevent possible concatamer formation of PCR products. Indeed, these modifications reduced the occurrence of “mixed fragments” to negligible levels (less than 0.00025% of reads) in the CRoPS run reported here.

Although it was attempted to carefully pool AFLP products of both samples in equal amounts, a somewhat skewed sample distribution in terms of reads per sample (39% sample 1 and 61% sample 2) was obtained. With varying levels of deviation from equal sample representation, this has also been observed in at least six other GS 20 runs (data not shown), despite our attempts to pool equimolar amounts of AFLP products. Clearly it would be beneficial to reach more equal sample representation to increase the number of SNPs mined per run. The same observation was made by Binladen and co-workers [27]. In their study the effect of 5′-tag sequences was suggested as a likely explanation for the single molecule sequence variations. We support this explanation as we have observed in this GS 20 run and other subsequent runs (data not shown) that 5′ tag sequences containing “AC” bases at the 5′ end are significantly underrepresented when equimolar amounts of tagged sample DNAs are pooled. Therefore, in retrospect the choice of a sample identification tag containing “5′-AC” has contributed to the observed skewed sample distribution.

Other optimization steps expected to increase the output of CRoPS further include selection against plastid (chloroplast sequences) co-isolated with genomic DNA, the use of normalized genomic or cDNA [28] libraries or other methods of enrichment for unique, single-copy sequences such as High C_o_t selection [15] or methylfiltration [16], prior to AFLP amplification. The use of such enrichment methods contribute to losing as little as possible sequence capacity to (highly) repeated sequences. Obviously, the output of a CRoPS run will also increase considerably as a result of the recent introduction of the GS FLX which has output specifications of over 400,000 reads with average read length of 240 nt. The increased read length does not only increase the amount of basepairs per run but also reduces the fraction of SNPs that can not be exploited due to insufficient flanking sequence information available for assay development. In conclusion, CRoPS is a powerful technology for random genetic marker development, which meets the shortcomings intrinsic to many plant species, i.e. the lack of available sequence information, large genomes containing high proportions of duplicated sequences and/or low levels of polymorphism. In the absence of whole-genome draft sequences, high-throughput sequencing of genome representations of multiple accessions in parallel using CRoPS will supply sufficient genetic (single nucleotide) polymorphisms to allow marker-assisted selection using existing genotyping platforms. It is our expectation that these developments will allow high-resolution sequence-based breeding using thousands of genetic markers to become reality in the nearby future.

## Materials and Methods

### AFLP target preparation

Total genomic DNA was isolated from leaf material of the two parental lines (i.e. B73 & Mo17) of the ISU mapping population (www.maizegdb.org), using a modified CTAB procedure [29]. These 2 parental lines were chosen to be able to validate and map the discovered SNPs in the ISU mapping population.

AFLP templates were prepared as described previously [20]. In short, 100–500 ng total genomic DNA was digested using 5 units *Hpa*II and 2 units *Mse*I for at least 1 hour at 37°C. After digestion, the mixture was heated at 80°C for 10 min. Next, AFLP adapter ligation using *Hpa*II and *Mse*I adapters was carried out for 3 hours at 37°C. The restriction-ligation (RL) mixture was subsequently diluted 10-fold with T_10_E_0.1_ and 5 µl diluted mix was used as a template in a selective pre-amplification step, the so-called +1/+1 pre-amplification. Primer sequences for the +1/+1 pre-amplification were 5′-GTAGACTGCGTACACGGA-3′ (*Hpa*II site, including 1 selective nucleotide “A”) and 5′-GATGAGTCCTGAGTAAC-3′ (*Mse*I site, including 1 selective nucleotide “C”). Twenty µl PCRs were performed containing 5 µl diluted RL mixture, 30 ng *Hpa*II primer, 30 ng *Mse*I primer, 0.2 mM dNTP, 0.4 U AmpliTaq® (Applied Biosystems) and 1× AmpliTaq buffer. PCR was performed for 20 cycles with the following cycle profile: 30 sec 94°C, 60 sec 56°C, 60 sec 72°C, followed by cooling down to 4°C.

The +1/+1 pre-amplification reaction was diluted 20-fold with T_10_E_0.1_, and used for the second selective amplification step, the so-called +1/+2 selective amplification. Primer sequences for the +1/+2 selective amplification were 5′-‘P-ACACGTAGACTGCGTACACGGA-3′ (*Hpa*II site, including 1 selective nucleotide “A”) and 5′-‘P-ACACGATGAGTCCTGAGTAACT-3′ (*Mse*I site, including 2 selective nucleotides “CT”) for sample B73. The four most 5′ bases of these primers serve as sample identification tag (KeyGene™ SeqTag technology). These 4-nt sample identification tags were selected from a collection of 4-nt sequences differing by at least 2 nt to exclude the possibility that a single nucleotide substitution error could cause incorrect assignment of the sequence to a sample. Similarly, primer sequences for the +1/+2 selective amplification of the Mo17 sample were 5′-‘P-AGCTGTAGACTGCGTACACGGA-3′ (*Hpa*II site, including 1 selective nucleotide “A”) and 5′-‘P-AGCTGATGAGTCCTGAGTAACT-3′ (*Mse*I site, including 2 selective nucleotides “CT”). Fifty µl PCRs were performed containing 5 µl diluted +1/+1 pre-amplification mixture, 75 ng *Hpa*II primer, 75 ng *Mse*I primer, 0.2 mM dNTP, 1 U AmpliTaq (Applied Biosystems) and 1× AmpliTaq buffer. PCR was performed for 30 cycles with the following cycle profile: 30 sec 94°C, 60 sec 56°C, 60 sec 72°C, followed by cooling down to 4°C.

Next, 100 µl of PCR products of each sample were purified using the QIAquick PCR Purification Kit (Qiagen). Concentrations of both samples were determined using the Nanodrop ND-1000 (Nanodrop Technologies), after which equal amounts of the two samples were pooled and further treated as one fragment library sample. This saves costs and prevents relying on physical compartmentalization to separate both samples. Furthermore this approach provides flexibility regarding processing multiple samples.

### GS 20 library preparation & titration

3.45 µg of the fragment library sample (i.e. pooled, purified and tagged AFLP products) were used as input for GS 20 library construction. The use of tagged and pooled PCR products, however, necessitated several adaptations in the published GS 20 library construction protocol [23]. First, no shearing was carried out. Second, the end-polishing step was omitted, and modified A and B adapters were used as follows: adapter A-upper strand: 5′-CCATCTCATCCCTGCGTGTCCCATCTGTTCCCTCCCTGTCTCAGT-3′, adapter A-lower strand: 5′-CTGAGACAGGGAGGGAACAGATGG-3′, adapter B-upper strand: 5′-BIO-TEG-CCTATCCCCTGTGTGCCTTGCCTATCCCCTGTTGCGTGTCTCAGT-3′ and adapter B-lower strand: 5′-P-CTGAGACACGCAACAGGGGATAGGCAAGGCACACAGGGGATAGG-3′. Finally, the *Bst* DNA polymerase fill-in step of the published protocol was left out.

After library construction, a titration run was carried out using 16, 64, 256 and 512 copies per bead. The copies per bead ratio to be used in the titration run is estimated based on the concentration of sstDNA (single stranded AB library). Therefore, the outcome of the titration run determines the ratio which needs to be applied in the actual sequencing run. Based on the titration run carried out for this experiment, a 48 copies per bead ratio was selected, founded on the “Predicted Recovery” of approximately 2.10^6^ enriched beads, > 60% PassFilter, <20% Mixed+Dots and approximately 6000 Keypass reads.

### GS 20 sequencing

Emulsion PCR and bead enrichment were carried out according to the standard GS 20 protocol (Roche Applied Science). One full picotiterplate (PTP) (70×75 mm) with two regions was used. Enriched beads were divided over both regions. Sequencing was performed according to the manufacturer's instructions (Roche Applied Science).

### CRoPS bioinformatics pipeline

The basecalled reads from both regions were added together in one file and further processed for SNP mining using a fully automated pipeline (Keygene N.V.). The web based pipeline was written in Perl 5.8.0 and runs via an Apache web server on a Linux platform. Microsoft Internet Explorer was used as client. An Oracle 10*g* relational database served as the central repository for all raw and processed data and the material and process definition.

The SNP discovery process consisted of four parts, namely (1) GS 20 data processing, (2) CRoPS pre-processing, (3) the CRoPS analysis, and (4) CRoPS SNP mining (Fig. 1).

GS 20 data processing was performed on-rig using the standard GS software. This process resulted in the “GS 20 raw sequence reads” that were directly used for further processing in the CRoPS pre-processing step. During pre-processing, the origin of the reads was identified according to their four base sample identification tags. The implementation of this step was based on the internal BLAST function in Oracle 10*g* (Oracle). Furthermore, the AFLP adapter sequences were trimmed. Pre-processed reads were saved to the database. In the CRoPS analysis step, reads were clustered and assembled using the TGICL tool [26]. Clustering was performed using the following variable parameters: minimum percent identity for overlaps (94%), minimum overlap length (30 nt) and maximum length of unmatched overhangs (30 nt). Again, all data obtained were subsequently saved to the Oracle database.

Polymorphisms were selected during the SNP mining step. For each putative SNP a number of features were recorded, including relative position in the consensus sequence, sample count in the MSA, allele count per sample, the MSA depth (number of reads at the SNP position), distance to flanking SNPs, flanking size around SNP and the presence of a homopolymer stretch in which a SNP may occur. Mining rules were created from these features and defined as follows: 1) each allele should be present at least twice in a MSA, 2) SNPs should not be part of homopolymers larger than 3 bases, and 3) SNPs should be segregating according to sample origin. SNPs that passed the filters were selected as the best candidates for conversion into genotyping assays.

The pre-processed sequence data will be deposited at the NCBI Short Read Archive (SRA) as soon as this archive is ready to accept the data (expected at the end of 2007). Until then, the data can be requested from the authors.

### SNPWave

Probes were designed for 30 putative SNPs in two multiplex (15-plex) SNPWave assays using ProbeDesigner software (Keygene N.V.) as described previously [24]. SNPWave reactions were carried out as described previously [24]. In short, ligation reactions were carried out in 10 µl volume containing 200–400 ng total genomic DNA, 1×Taq DNA ligase buffer [20 mM tris-HCl, 25 mM KAc, 10 mM MgAc_2_, 10 mM dithiothreitol (DTT), 1 mM NAD, 0.1% triton X-100; pH 7.6 at 25°C; New England Biolabs Inc], 2 U Taq DNA ligase (New England Biolabs Inc) and 0.5 fmol of each of the ligation probes. Next, 10 cycles of repeated denaturation, probe hybridization and ligation were performed in a Perkin Elmer 9700 thermal cycler (Applied Biosystems) using the following profile: initial denaturation for 2 min at 94°C, followed by 10 cycles of 15 s at 94°C and 60 min at 60°C, and storage at 4°C. After ligation, the mixture was diluted with 30 µl of 1×Taq DNA ligase buffer to 40 µl.

Ten µl of diluted ligation reaction was subsequently amplified in a 20 µl mixture containing 1×GeneAmp® PCR buffer (Applied Biosystems), 0.2 mM of each dNTP, 0.4 U AmpliTaq Gold DNA polymerase (Applied Biosystems), and 30 ng unlabeled forward primer (5-GACTGCGTACCAATTC-3) and 30 ng FAM-labeled reverse primer (5-GATGAGTCCTGAGTAA-3). The amplification profile was 12 min at 94°C, followed by 13 cycles of 30 s at 94°C, 30 s at 65°C with a reduction of 0.7°C per cycle to 56°C in cycle 13, followed by 1 min at 72°C. This was followed by 23 cycles of 30 s at 94°C, 30 s at 56°C and 1 min at 72°C, and storage at 4°C.

Purification of diluted SNPWave PCR products and subsequent detection on the MegaBACE 1000 (Amersham Biosciences/GE Healthcare Life Sciences) were as described previously [24].

SNPs and flanking sequences can be found in the supplementary file (Table S1). Probe sequences are available upon request from the authors.

## Supporting Information

Table S1 SNPs and flanking sequences used for the 13-plex and 10-plex SNPWave assays
(0.04 MB DOC)
