# Supplementary material for: Complexity Reduction of Polymorphic Sequences (CRoPS™): A Novel Approach for Large-Scale Polymorphism Discovery in Complex Genomes
Source: PLoS One. 2007 Nov 14;2(11):e1172. doi: 10.1371/journal.pone.0001172 (PMC2048665; doi:10.1371/journal.pone.0001172)
Supplement: Table S1 — SNPs and flanking sequences used for the 13-plex and 10-plex SNPWave assays (0.04 MB DOC) [file pone.0001172.s001.doc]

Table S1: SNPs and flanking sequences used for the 13-plex and 10-plex SNPWave assays

| **Loci** | **Fragment sequence** |
| --- | --- |
| **13-plex SNPWave assay** | |
| 1 | CCGGAGAAGGCCTCGGCAAGGCTCT**[G/C]**TGGGTCCAGCAGGTCGGCGCGGAGGAGGACGAGGCGGTCG |
| 2 | TTCTTCGT*ATATACATGCATGGAGAAGATGCCAATTGGT**[A/C]**TATTGGAGGCTCTTCCGATCC*CTGGTGGGCCTGGTCGAT |
| 3 | TTAACTGCCCTGCTCCTGCTGGTCCTTGTTGG**[T/G]**AGTCCAAAA*TGGTCGTGCTCTGCTGTTA*TTATGGCTAT |
| 4 | CCGGATCTCTGGAGACCGATAG**[T/A]**GAGTCTGTACATGGCATGGCTAGCTTGGAACAAGTCTCAT |
| 5 | TTAACTACCCTGCCATGCCCATT**[A/G]**GCGGAATAACCGAAGAACATGACGCAGAGAGCGCCAGGAA |
| 6 | TGTCTGCTGTGGTCCGCCCACGGACGGACGCCG**[C/T]**AGTGCTGTCCAGAGCGGTTTGCCCGTCTGGTCTGGACGGG |
| 7 | T*GCTAAAAAA*CAATTTGAGATATAACGCGACAATGTAC**[A/G]**TGTACGGAGTCATGAAGAAGCTTGGGGAAAG |
| 8 | CTCTTGGACAGTCCACAGCCTCCTACAAAGCCGCTGCTCG**[G/T]**CTTGCGAGTGCGACTCCACCTCCGG |
| 9 | CCGGACCCCACATTATAT**[A/G]**GGCTACAGGACGGGCCTTAGTAGTTCCCTTTTGGAGTGTA |
| 10 | ACGTTTTAGTTACCTGCTCTAGGATGATAAGAGGTTTGGG**[T/G]**ATCTCATGGCGACCGAGGAGCGCAGTTCCTTTGTAGTTAA |
| 11 | CTGTTTGGTTGAAAGCCT*GCCAGACAATTGACTGCCAGG**[T/C]**ACACAATCTCAACCCCAGGCGA |
| 12 | CGTACACGGATCGATCGAGCGG**[A/G]**ACGGAACATGCACTTGCTTTGCGTTGCGCCCGCGCGCGCG |
| 13 | ACTGCGTACACGGAAGGGGGTCATTCATACTCATCATA**[T/C]**AGCAGGGAAGACGTGGGCCCAAATCTGCCCGCTCGGAGTT |
|  | |
| **10-plex SNPWave assay** | |
| 1 | CCGGACCCACGGGCCGCGGACGAGGTCA**[C/G]**GTCAGTGATCAGTGACTTCAGTTTAGTTAA |
| 2 | CCGGACAAACACAACCAAAAGTACACTC**[A/G]**CCATCAGAGCCATTTTCTTTTGCCGAATTGATTTGATGGG |
| 3 | CCGGACACCGCGGCGGCGACTGCAA**[A/G]**GAAAAGGATGGGGAGCAACGAGAGCTTGAAGCTTTCCATG |
| 4 | TTAACTGCAGTGCTGAAGTATGCTA*TCGC**[A/G]**CTTTCTTTTCCTT*CAGGTGAGAAACT*GAAAGTTGACGC |
| 5 | TTCTCGCAT*GATTTGCTCCGTAGCCATGTGACCCCTACC**[A/T]**AAGGCCAAAGCACCACGTCGGCGGGCGCAAGCG |
| 6 | ACATTCTGCCGTGCAATGTTGAGAGCAAGATGTGTCAACA**[C/G]**TTACATTTTGTCTTGCATTCAAAAGA |
| 7 | TCTGCCCAAAGATCCACATTGCGATGTTTAGGACCGAGGA**[G/T]**GGAAGCTATGCATCACCTTGTTTG |
| 8 | CCGGATCTACACCGCCGCCGC**[A/G]**GGATTATGAGCTGCTGGGTCCAAATGGGGCCATACACATG |
| 9 | AGATCCTTGTGCCTTCCCAGTCCAGCACCTCGACACATGC**[G/T]**TCAGACTTCATCTCCACCTACAT |
| 10 | CCGGAGATAGCCTTATCGACGATGAGTGTCATTGATGG**[C/T]**GACGGGTTATAATAATGTTAGGTCAATAATGACAAAAGTG |
